# First identification of *Cryptosporidium parvum* subtype IIaA15G2R1 and two *Eimeria* species in the edible dormouse (*Glis glis* Linnaeus, 1766)

**DOI:** 10.1007/s11259-026-11197-1

**Published:** 2026-04-07

**Authors:** Seila Couso-Pérez, Xosé Pardavila, Ramsés Pérez-Rodríguez, Elvira Ares-Mazás, Hipólito Gómez-Couso

**Affiliations:** 1 Laboratory of Parasitology, Paraquasil Group (GI-2109), Department of Microbiology and Parasitology, Faculty of Pharmacy, Universidade de Santiago de Compostela, Santiago de Compostela, A Coruña 15782 Spain; 2 Aquatic One Health Research Center, Universidade de Santiago de Compostela, Santiago de Compostela, A Coruña 15782 Spain; 3 Sorex Ecoloxía e Medio Ambiente S.L., Santiago de Compostela, A Coruña 15702 Spain; 4 Asociación para a Defensa Ecolóxica de Galiza (ADEGA), Santiago de Compostela, A Coruña 15704 Spain

**Keywords:** *Glis glis*, *Cryptosporidium*, *Eimeria*, Wild rodent

## Abstract

As part of the edible dormouse (*Glis glis* Linnaeus, 1766) monitoring program, faecal samples of seven animals from Montes do Invernadeiro Natural Park (Galicia, NW Spain) were collected following current guidelines for the ethical use of animals in research. Fresh faecal samples were individually processed using a diphasic concentration method and nucleic acids were extracted. PCR protocols were applied to amplify a fragment of the small-subunit ribosomal RNA (*ssu-rRNA*) gene of *Eimeria* as well as fragments of *ssu-rRNA* and 60-kDa glycoprotein (*gp60*) *loci* of *Cryptosporidium* spp. The hypertransmissible *Cryptosporidium parvum* subtype IIaA15G2R1 was identified in one of the seven samples analysed (14.3%). Partial sequences of the *Eimeria* spp. *ssu-rRNA* gene were obtained from all seven faecal samples (100%), identifying *Eimeria jerfinica* and *Eimeria cahirinensis* in one and six samples (14.3 and 85.7%, respectively). This work reports for the first time the occurrence of *Cryptosporidium* and two *Eimeria* species in glirid hosts, specifically in edible dormouse (*G. glis*), demonstrating the widely distribution of these parasites in wildlife. The identification of the zoonotic *C. parvum* subtype IIaA15G2R1 revealed that this glirid may serve as a reservoir of cryptosporidiosis. The identification of *E. jerfinica* and *E. cahirinensis* highlights the need for further studies to clarify the nature of these findings.

**Supplementary Information:**

The online version contains supplementary material available at 10.1007/s11259-026-11197-1.

## Introduction

The edible dormouse (*Glis glis* Linnaeus, 1766) is a nocturnal and arboreal glirid rodent. Its distribution includes most of Europe and the Caucasus, eastern Turkey and northern Iran. A narrow area in the north of the Iberian Peninsula represents the south-western border of this rodent (Holden-Musser et al. 2016). In Galicia (NW Iberian Peninsula), the edible dormouse was reported for the first time in 19th century by López-Seoane (Ortiz-Jiménez and Pardavila 2021) and it has currently been described in two isolated mountain areas, O Courel and Montes do Invernadeiro. The edible dormouse inhabitis mainly dense, mature and well-preserved mountain forests, in which beech and oak species predominate (Ortiz-Jiménez and Pardavila 2021; SECEM 2025). The diet of this rodent is predominantly herbivorous and consists almost exclusively of acorns, beechnuts and hazelnuts, and blackberries sometimes (Ortiz-Jiménez and Pardavila 2021). During the active period, these animals take refuge in tree hollows and in nest boxes designed exclusively for them, which are used in studies of population structure and dynamics. Specifically in Montes do Invernadeiro (Galicia, NW Spain), the occupancy rate of the nest boxes was 56–96% between 2016 and 2019 (Ortiz-Jiménez and Pardavila 2021). The edible dormouse is included in the International Union for Conservation of Nature category of low concern. However, in Galicia, the replacement of leafy and scrubby vegetation with coniferous and eucalyptus forests, together with the frequent fires in the region, pose significant threats that could negatively affect its conservation (Ortiz-Jiménez and Pardavila 2021).

Members of the genus *Cryptosporidium* and *Eimeria* (Apicomplexa) are obligate protozoan parasites that infect the cells of digestive tract of numerous vertebrates (mammals, birds, reptiles, amphibians and fish) (Duszynski and Upton 2001; Ryan et al. 2021). They have direct life cycles, completing their development within a single host species. *Eimeria* spp. oocysts become infectious after environmental sporulation, whereas *Cryptosporidium* spp. oocysts are already infectious when they are shed in host faeces (Duszynski and Upton 2001; Ryan et al. 2021).

There are no previous studies on the prevalence or molecular characterization of *Cryptosporidium* spp. in glirids reported in the scientific literature, and only one *Eimeria* species, *Eimeria gliris* (Musaev and Veysov 1961), has been described in the edible dormouse. In this brief report, we present for the first time the molecular data obtained on these parasites in faecal samples of edible dormouse (*G. glis*).

## Materials and methods

### Study area, sample collection and processing

**Fig. 1:**
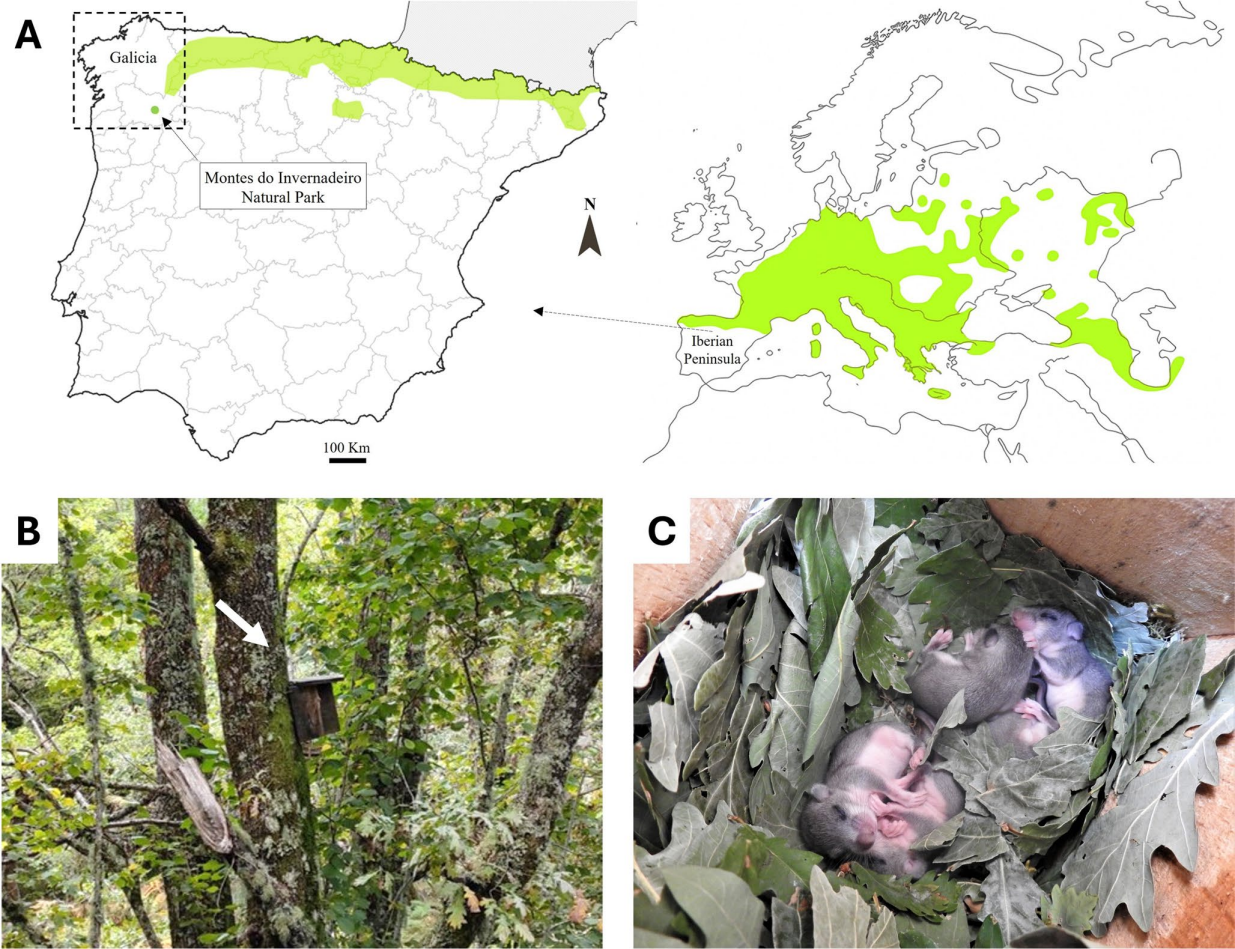
**A**, worldwide distribution of edible dormouse (*Glis glis*) and enlargement of the Iberian Peninsula showing the region of Galicia (NW Spain) and the sampling area (green dot). **B**, location of a wooden nest box (arrow) in a tree trunk from the *G. glis* conservation program in the Montes do Invernadeiro Natural Park. **C**, litter of *G. glis* among oak leaves inside a wooden nesting box

In September 2020, as part of an edible dormouse monitoring programme in Montes do Invernadeiro Natural Park (42º07´52´´N 7º19´09´´W; Galicia, NW Iberian Peninsula; Fig. 1A; Fig. S1), a protected natural and practically human uninhabited mountainous area, seven edible dormice (*G. glis*) (one male and six females) with body weight 108.8 ± 36.9 g were caught during their diary resting activity in wooden nest boxes placed in tree trunks (Fig. 1B and C). The animals showed no apparent signs of disease, and their capture was conducted in accordance with current guidelines for the ethical use of animals in research.

Fresh faecal samples were collected directly from each specimen immediately after capture and stored at 4 °C until analysis. They were then processed as previously reported (Couso-Pérez et al. 2022). Briefly, the faecal samples (4–5 pellets with formed consistency) were individually homogenized in a mortar with 30 mL of 0.04 M phosphate buffered saline (PBS) pH 7.2, filtered through a set of two sieves (150 and 45 μm of mesh size), shaken vigorously with diethyl ether (2:1) and concentrated by centrifugation at 1250 ×*g*, 4 °C for 15 min. The supernatant was carefully removed, and the sediments were resuspended in 1 mL of PBS 0.04 M pH 7.2 and stored at -20 °C.

### Molecular characterization

Nucleic acids were extracted from 200 µL aliquots of the sediments using the Stool DNA Isolation Kit (Norgen, Biotek Corp., Thorold, ON, Canada) according to the manufacturer’s instructions. Then, PCR protocols were used to amplify a ⁓420-bp and a ⁓587-bp fragments of the small-subunit ribosomal RNA (*ssu-rRNA*) of *Eimeria* and *Cryptosporidium*, respectively (Ryan et al. 2003; Couso-Pérez et al. 2019a). Moreover, a ⁓850-bp fragment of 60-kDa glycoprotein (*gp60*) *locus* of *Cryptosporidium* was amplified for subtyping purposes (Alves et al. 2003). Positive PCR products were purified and sequenced in both directions using the Sanger method by Eurofins Scientific (Luxembourg). Sequence data were analysed using SeqMan™ 7.0 software (DNASTAR^®^, Madison, WI, USA) and the derived consensus sequences were compared with other *Cryptosporidium* and *Eimeria* sequences deposited in the GenBank^®^ database via the public web interface of the BLAST^®^ 2.12.0 program (Sayers et al. 2021). Phylogeny analyses were conducted using MEGA X software (Kumar et al. 2018).

## Results

**Fig. 2:**
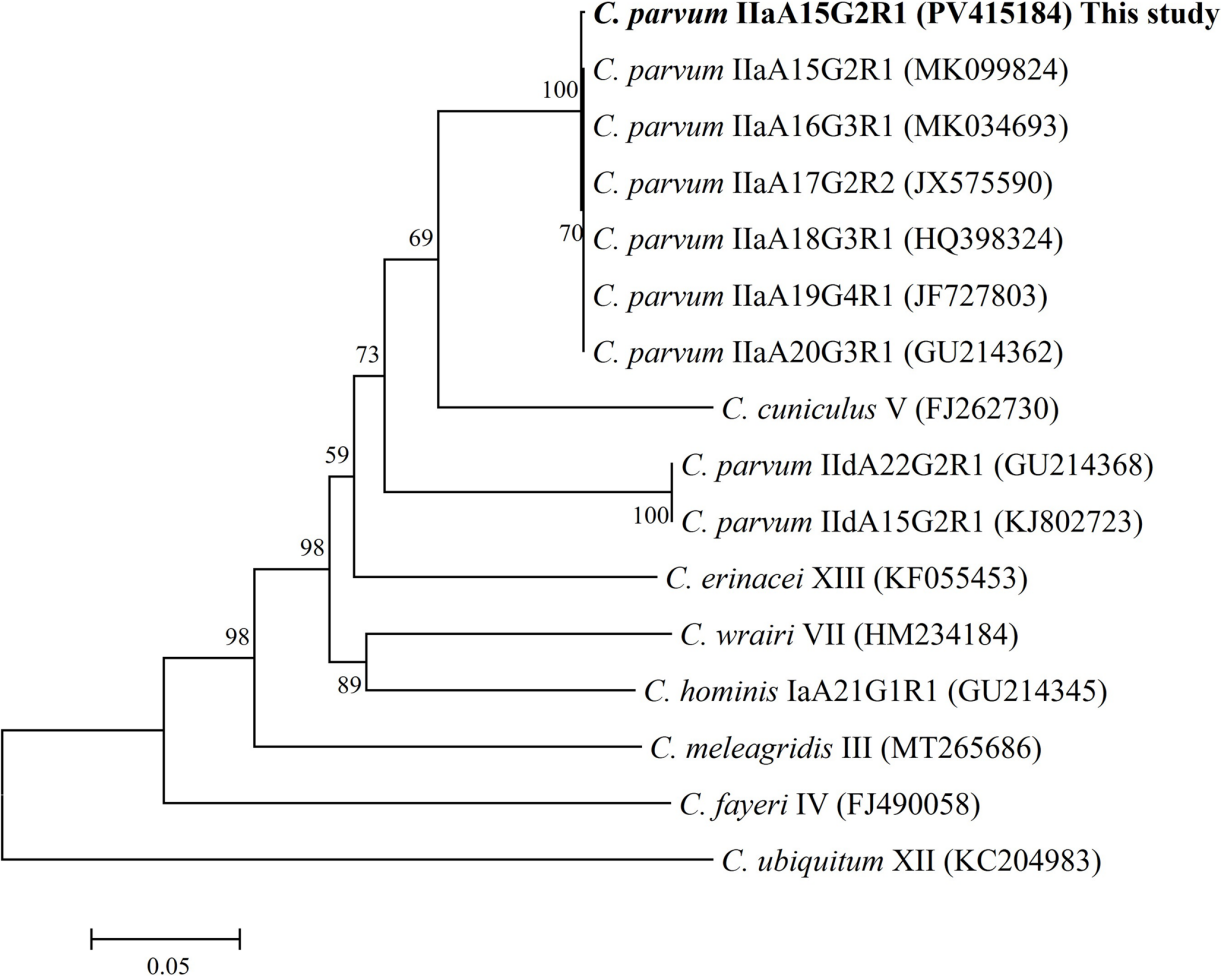
Phylogenetic relationships between the *Cryptosporidium* isolate from edible dormouse (*Glis glis*) and other *Cryptosporidium* spp. inferred by neighbour-joining analysis of a fragment of the 60-kDa glycoprotein (*gp60*) gene on the basis of genetic distances calculated by the Tamura 3-parameter model using MEGA X software. The tree was generated using a total of 638 positions in the final dataset. The percentages of replicate trees in which associated taxa clustered together in the bootstrap test (1,000 replicates) are shown at the internal nodes for distance (> 50%). Isolate obtained in this study is highlighted in bold type

By analysis of the *ssu-rRNA* of *Cryptosporidium*, a fragment of the expected size was amplified in one of the seven samples of *G. glis* analysed (14.3%). The subsequent PCR amplification and sequencing of a fragment of the *gp60* gene of *Cryptosporidium* allowed the identification of *Cryptosporidium parvum* subtype IIaA15G2R1 (GenBank^®^ accession number: PV415184). The *gp60* partial sequence showed 99.8% homology with respect to sequence with the accession number MK099824 (Fig. 2).

**Fig. 3:**
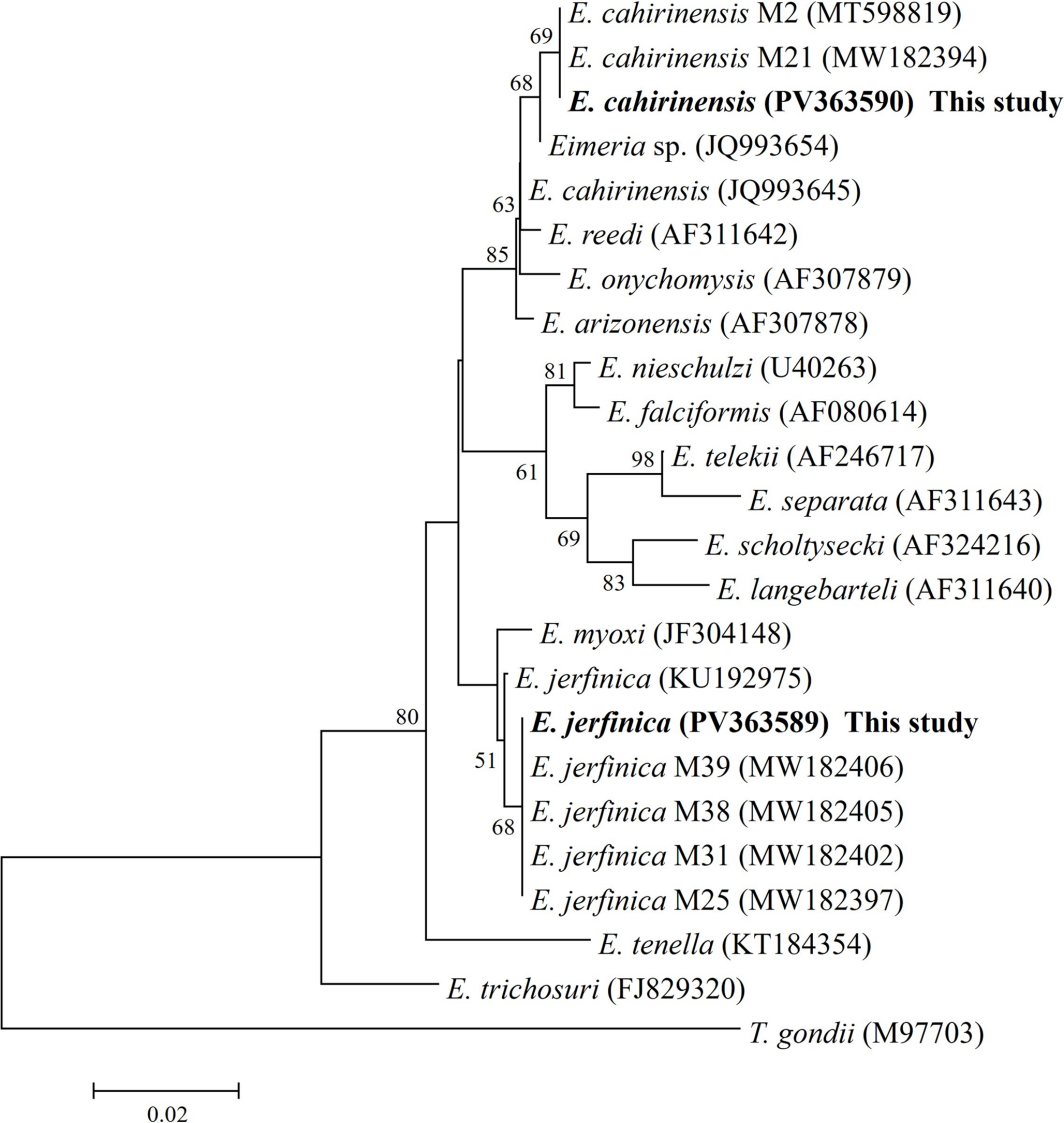
Phylogenetic relationships between *Eimeria* isolates from edible dormouse (*Glis glis*) and other *Eimeria* spp. inferred by neighbour-joining analysis of a fragment of the small-subunit ribosomal RNA (*ssu-rRNA*) gene on the basis of genetic distances calculated by the Tamura-Nei method using MEGA X software. The tree was generated using a total of 363 positions in the final dataset. The percentages of replicate trees in which associated taxa clustered together in the bootstrap test (1,000 replicates) are shown at the internal nodes for distance (> 50%). Isolates obtained in this study are highlighted in bold type

Partial sequences of the *ssu-rRNA* gene of *Eimeria* were obtained from all seven faecal samples (100%). A rodent-specific *Eimeria* species, *E. jerfinica*, was identified in one edible dormouse (14.3%; GenBank^®^ accession number: PV363589) with a similarity of 99.7% with respect to the sequence KU192975 deposited in the GenBank^®^ database. In the remaining six samples (85.7%), the same *Eimeria cahirinensis* sequence was identified (GenBank^®^ accession number: PV363590), which showed 100% identity with the sequence deposited with accession number MW182394 (Fig. 3).

## Discussion

The present study provides insights into protozoan parasites in poorly studied rodent species, despite the limited sample size associated with the protected status of *G. glis* and the strict ethical regulations governing its capture. Furthermore, the low population density and fragmented distribution of this species in Galicia restrict the possibility of sampling a larger number of individuals without compromising animal welfare or habitat conservation. Nevertheless, this study reports for the first time the occurrence of *Cryptosporidium* and two *Eimeria* species in glirid hosts, specifically in the edible dormouse (*G. glis*), and analyses their phylogenetic relationships.

To our knowledge, this is the first time that the presence of *Cryptosporidium* in glirid hosts has been investigated. Therefore, the detection of *C. parvum* in the edible dormouse (*G. glis*) extends the range of hosts for which this species has been identified. Furthermore, the hypertransmissible and virulent subtype IIaA15G2R1 was identified, which is reported as one of the predominant subtypes responsible of diarrhoeal disease in neonatal calves and zoonotic cryptosporidiosis worldwide (Feng et al. 2018). Previous studies conducted in the same geographical area showed that this genotype is not only the most prevalent in domestic ruminants but has also been identified in a wide range of hosts, including wild ponies from the highland areas of the northern Iberian Peninsula and brown trout (*Salmo trutta*) captured in Galician rivers (Díaz et al. 2010; Couso-Pérez et al. 2019b, 2020).

Regarding the identification of *E. jerfinica*, this species was described in the wood mouse (*Apodemus sylvaticus*) in Azerbaijan (Musaev and Veysov 1961) and recently it was molecularly detected in several rodent species (*Apodemus agrarius*, *Apodemus flavicollis* and *A. sylvaticus*) from several regions in Europe (Mácová et al. 2018). Although *E. jerfinica* has not been reported in rodents in the Iberian Peninsula, its presence was indirectly revealed in a previous study conducted on Spanish bats (Couso-Pérez et al. 2022). Thus, it was identified in 25 samples from 11 of the 32 chiropteran species inhabiting the Iberian Peninsula, and specifically in four bat species from the same geographical area in which this study was conducted (GenBank^®^ accession numbers: MW182397, MW182402, MW182405 and MW182406; Fig. 3). It was suggested that this may be a spurious infection acquired through the food chain, or via direct contamination by faeces from infected rodents when sharing roosting sites with other animals (Couso-Pérez et al. 2022). Studies have shown that edible dormice can be displaced from their nest boxes by other species, including yellow-necked mice (*A. flavicollis*), wood mice (*A. sylvaticus*), Leisler’s bats (*Nyctalus leisleri*), black rats (*Rattus rattus*) and bird species of the Troglodytidae family, which may pose a risk of infection to edible dormice (Ortiz-Jiménez and Pardavila 2021). The first identification of *E. jerfinica* in *G. glis* in the present study supports previous findings obtained from bats, thereby extending the range of hosts for this rodent coccidian parasite.

The consensus sequence of *E. cahirinensis* obtained in six faecal samples from *G. glis* was 100% identical to *E. cahirinensis* previously detected in bats in Galicia, specifically in a lesser horseshoe bat (*Rhinolophus hipposideros*) from the same natural park where this study was conducted, as well as in faecal samples of greater horseshoe bat (*Rhinolophus ferrumequinum*) (sequences deposited in the GenBank^®^ database under accession numbers MW182394 and MT598819, respectively). Moreover, the sequence identified in the edible dormouse in the present study showed 99.8% similarity to *Eimeria* sp. isolated from a spiny mouse of the genus *Acomys*, and 99.5% identity to *E. cahirinensis* found in *Acomys* cf. *cahirinus* (sequences deposited under accession numbers JQ993654 and JQ993645, respectively) (Fig. 3). The identification of sequences almost identical to *E. cahirinensis*, traditionally associated with the genus *Acomys* (a rodent widely distributed across the African continent), could likely be attributed to current limitations in genetic databases or the existence of closely related, yet undescribed, *Eimeria* species specific to glirids that share high genetic similarity at the analysed *locus*. However, other less probable pathways for the presence of this coccidian parasite should not be ruled out. Although *Acomys* is not endemic to the Iberian Peninsula, its availability in pet shops and the potential for accidental introductions ‒facilitated by geographical proximity and maritime traffic with the African continent‒ could represent alternative explanations, albeit less parsimonious, for the presence of these sequences in *G. glis* from Montes do Invernadeiro Natural Park (Kvičerová et al. 2007). Although *Eimeria* species are the most frequently detected coccidia in rodents (Levine and Ivens 1990) with more than 400 species morphologically described in these hosts (Duszynski and Upton 2001), molecular data are very scarce. Currently available data are from 22 *Eimeria* species infecting 11 rodents, thereby further studies of morphological, histological, and molecular characterisation of *Eimeria* infections in rodent hosts are required.

## Conclusions

This study is the first reporting the occurrence of *Cryptosporidium* and two *Eimeria* species in glirid hosts, specifically in the edible dormouse (*G. glis*) in the Iberian Peninsula. This demonstrates the distribution of these parasites in free-living animals. The identification of the zoonotic *C. parvum* subtype IIaA15G2R1 highlights the possible role of glirids in the transmission of sylvatic cryptosporidiosis, revealing that this glirid may serve as a reservoir of this zoonotic disease. In addition, the identification of *E. jerfinica* extends the host range and its geographical distribution. Furthermore, the detection of *E. cahirinensis*, which is specific to spiny mice (genus *Acomys*), emphasises the need for further research to clarify the nature of these findings.

## Supplementary Information

Below is the link to the electronic supplementary material.

Supplementary Material 1 (DOCX 3.07 MB)

## Data Availability

No datasets were generated or analysed during the current study.
