# Supplementary material for: First identification of Cryptosporidium parvum subtype IIaA15G2R1 and two Eimeria species in the edible dormouse (Glis glis Linnaeus, 1766)
Source: Vet Res Commun. 2026 Apr 7;50(4):255. doi: 10.1007/s11259-026-11197-1 (PMC13056733; doi:10.1007/s11259-026-11197-1)

**First identification of *Cryptosporidium parvum* subtype IIaA15G2R1 and two *Eimeria* species in the edible dormouse (*Glis glis* Linnaeus, 1766)**

**Supplementary material**

**Figure S1.** Map of the Montes do Invernadeiro Natural Park (Galicia, NW Iberian Peninsula) with sampling locations established during the edible dormouse monitoring programme.


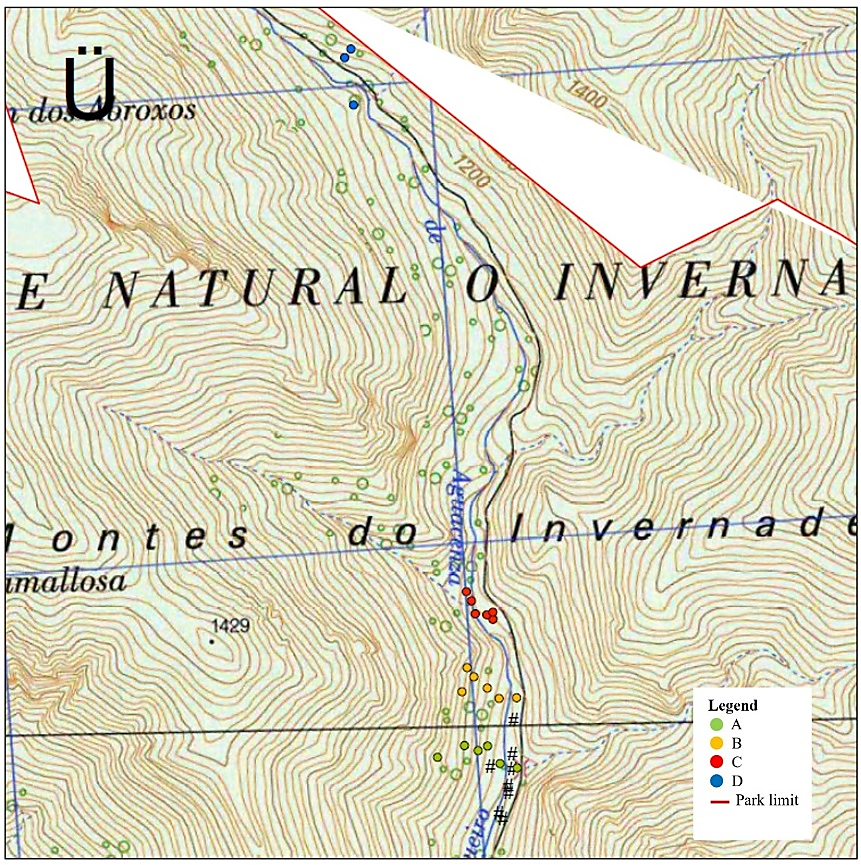


The edible dormouse monitoring programme was carried at four different locations of the Montes do Invernadeiro Natural Park, in which lines of nest boxes (A, B, C and D) were placed.
Map scale 1:10,000.

In this study, edible dormice (*Glis glis*) were captured at nest boxes located at points A (green) and B (yellow). The nest boxes were placed in trees at a height of 3-4 m above the ground and were separated from each other by 20-30 m. The faecal samples analysed in this study were obtained from individuals occupying different boxes, ensuring that the captured animals did not share nest sites.

**Figure S2.**


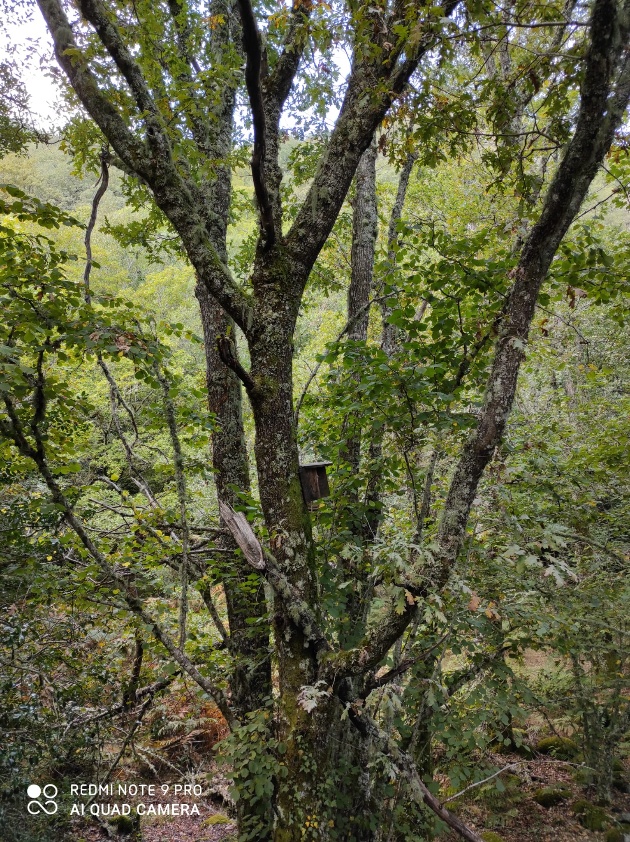

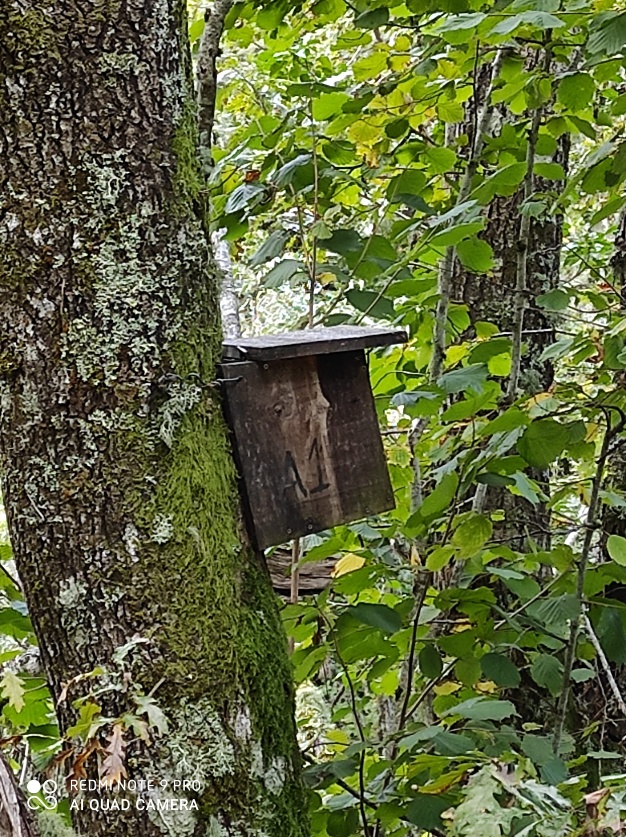

Supplement: Supplementary file 1 — Supplementary Material 1 (DOCX 3.07 MB) [file 11259_2026_11197_MOESM1_ESM.docx]
